# Effects of Silage Inoculants on the Quality and Microbial Community of Whole-Plant Corn Silage Under Different Fertilization Treatments

**DOI:** 10.3390/microorganisms14010065

**Published:** 2025-12-27

**Authors:** Deli Dong, Gulinigeer Ainizirehong, Maierhaba Aihemaiti, Xin Huang, Yang Li, Huaibing Yao, Yuanyuan Yan, Min Hou, Weidong Cui

**Affiliations:** Xinjiang Laboratory of Special Environmental Microbiology, Xinjiang Uygur Autonomous Region Academy of Agricultural Sciences, Institute of Microbiology, Urumqi 830091, China

**Keywords:** different fertilization treatments, silage inoculant, forage quality, microbial community

## Abstract

The purpose of this study is to investigate the effects of silage inoculants (FJ) and natural fermentation (CK) on the quality and microbial community of whole-plant corn silage under different fertilization treatments, including conventional fertilization (CK), liquid microbial inoculant and conventional fertilization (JJ), and microbial organic fertilizer and conventional fertilization (YJ). After 30 days of room-temperature fermentation, parameters including pH, LA, CP, starch, ADF, NDF, and the microbial community were determined. The results showed that after 30 days of ensiling, silage inoculants significantly affected the nutritional components and fermentation parameters of whole-plant corn silage under different fertilization treatments. Furthermore, the two factors (silage inoculants and different fertilization treatments) exhibited a significant interaction effect. Simple effects analysis revealed that the significant interaction was mainly driven by a more pronounced differential effect of fertilization treatments on the nutritional indicators (starch, CP, ADF, and NDF) under silage inoculant (FJ) addition than under natural fermentation (CK) (*p* < 0.05). Among all silage treatments, the silage inoculants + microbial solution drip irrigation and conventional fertilization (FJJJ) group exhibited relatively superior silage quality. Specifically, the FJJJ group had the lowest contents of pH, ADF, and NDF, along with the highest contents of lactic acid (LA) and ether extract (EE). The addition of silage inoculants under different fertilization treatments consistently increased the abundance and reinforced the dominance of *Lactobacillus* in the microbial community. This effect was most pronounced in the FJJJ group, which showed the highest relative abundance. In contrast, the relative abundance of genera such as *Pantoea*, *Acinetobacter*, *Klebsiella*, and *Pseudomonas* decreased significantly. In summary, appropriate fertilization treatments combined with the addition of silage inoculants contribute to enhancing the quality of whole-plant corn silage and improve the fermentation microbial community of the silage. These findings provide a theoretical basis for producing high-quality corn silage.

## 1. Introduction

In recent years, with the rapid global development and the improvement of living standards, the consumption of animal products has surged dramatically [1]. Consequently, the animal husbandry industry has expanded rapidly; however, feed production has struggled to sustain the development of animal husbandry. Due to its characteristics of high biomass, high energy content, and high starch content, whole-plant corn silage has occupied an indispensable position in animal husbandry [2].

While whole-plant corn silage is rich in nutrients, its nutritional components and silage quality vary due to a variety of factors, such as fertilization management during cultivation and storage methods [3]. Therefore, producing high-quality whole-plant corn silage is of crucial importance. Fertilization with different formulations can improve the yield and quality of crops while minimizing resource waste and environmental pollution [4]. For instance, Liu et al. [5] found that compared to the application of compound fertilizer at rates of 300 kg·ha^−1^, 450 kg·ha^−1^, and 700·kg·ha^−1^, applying 600·kg·ha^−1^ of compound fertilizer achieved the best results in improving the growth index and fresh weight of silage corn. As an important fertilization method, microbial organic fertilizer has been extensively studied for its role in promoting plant growth [6]. These beneficial bacteria in organic fertilizers primarily promote plant growth through various metabolic activities [7]. For example, Thuc et al. [8] increased the yield of waxy corn and watermelon by using microbial fertilizer and lime. Silage is a simple and efficient feed storage technology, which is used to prolong the storage time of feed to address feed shortage issues in spring and winter. Silage involves compacting and sealing fresh plants to isolate air and create an anaerobic environment. It leverages the fermentation of endogenous lactic acid bacteria to produce organic acids, lower the pH value, and inhibit harmful bacteria, thereby achieving long-term preservation of fresh plants and reducing nutrient loss [9]. However, the production of high-quality silage is often limited by a low endogenous population of lactic acid bacteria, which can result in poor fermentation quality and nutrient loss [10]. Meanwhile, factors such as silage substrate types and oxygen penetration also affect silage quality [11]. Therefore, adding inoculants (*Pediococcus acidilactici*, *Lactiplantibacillus paracasei* and *Lactoccoccus lactis*) during ensiling is essential, as they have been shown to improve fermentation parameters and enhance the nutritional value of silage [12]. Meanwhile, the application of inoculants can also reduce the content of biological macromolecules in silage [13]. Studies have shown that the addition of *Lactiplantibacillus brevis* reduces antibiotic resistance genes (ARGs) and viral distribution in high-moisture corn kernels, thereby improving silage quality [14]. Additionally, Wang et al. found that the addition of *Lactiplantibacillus buchneri* alone or its combination with *Lactiplantibacillus plantarum* helps improve the aerobic stability of silage and enhances the utilization efficiency of silage feed [15].

Microbial community is also a key factor in evaluating silage quality [16]. Silage fermentation is a microbially driven process, and the core of its quality lies in the metabolic activity and diversity of microorganisms [17]. Therefore, the bacterial community composition during the silage fermentation process is the key to ensuring the success of its storage [18]. Furthermore, the fermentation process is dynamic in nature. Understanding the succession pattern of the microbial community is essential to identify the key beneficial microorganisms responsible for enhancing silage quality. Guan et al. [19] found that adding *Lactiplantibacillus buchneri* and *Lactiplantibacillus rhamnosus* to silage corn changed the dominant bacterial species to *Lactiplantibacillus buchneri* and *Lactiplantibacillus rhamnosus*, and *Acetobacter fabarum* occupied a dominant position after aerobic exposure.

Based on the above background, this study proposes the following specific and testable hypotheses: There is a significant interactive effect between fertilization methods and silage inoculants. This significant interaction primarily arises because the differential effects of various fertilization treatments on nutritional indicators are more pronounced under the condition of added silage inoculant (FJ) compared to natural fermentation (CK). Concurrently, by shaping a bacterial community centered around beneficial lactic acid bacteria with enhanced metabolic functions, superior silage quality can be achieved in terms of fermentation parameters (such as lactic acid content and pH) and nutritional components (including Starch, CP, ADF, and NDF). Li et al. [20] found that adding *Lactiplantibacillus plantarum* during silage production could mitigate the adverse effects on mulberry silage quality caused by high nitrogen fertilizer application during the cultivation period, with these effects varying depending on the harvest season. The purpose of this study is to treat whole-plant corn under different fertilization methods (conventional fertilization, conventional fertilization and drip irrigation with bacterial solution, conventional fertilization and microbial organic fertilizer) with silage inoculants, so as to evaluate the effects on silage quality and microbial communities. High-throughput sequencing was used to analyze the correlation between the dynamics of bacterial communities and silage characteristics. This study aims to provide a theoretical basis for optimizing the production of high-quality whole-plant corn silage.

## 2. Materials and Methods

The whole-plant silage corn used in this experiment was Xinsiyu 12 (a silage corn variety). The silage inoculant was compounded with *Lactiplantibacillus plantarum* (60%), *Lactiplantibacillus buchneri* (30%), *Lactiplantibacillus casei* (5%) and cellulase (5%), with a viable count of 10^12^ CFU/g. The liquid microbial inoculant was composed of *Bacillus subtilis*, with a viable count of 10^12^ CFU/mL. The microbial organic fertilizer was made from raw materials including *Bacillus subtilis*, straw and spent mushroom substrate, with a viable count of 10^12^ CFU/g. All the aforementioned preparations and corn seeds were provided by the Microbiology Research Institute, Xinjiang Uygur Autonomous Region Academy of Agricultural Sciences. The silage inoculant, liquid microbial inoculant, and microbial organic fertilizer used in this study were subjected to viable bacterial count determination via the standard plate count method prior to application.

One-way Air Valve Solid-state Anaerobic Fermentation Bag (1 kg): Purchased from HebeiCangzhou Ounuo Plastic Packaging Co., Ltd. (Cangzhou, China).

pH Meter (Model: PHS-3C): Purchased from China Shanghai Yidian Scientific Instruments Co., Ltd. (Shanghai, China).

### 2.1. Experimental Design

Three fertilization treatments were established: (1) CK (conventional fertilization): 1.67 kg·ha^−1^ diammonium phosphate and 0.67 kg·ha^−1^ compound fertilizer (During the 90-day period following sowing, irrigation was applied nine times at 10-day intervals, with nitrogen fertilizer supplied through fertigation during the first three irrigation events.); (2) JJ: CK + 0.1 L·ha^−1^ microbial inoculant (During each irrigation event, the liquid microbial inoculant was applied at a rate of 0.1 L ha^−1^ via the irrigation water.); (3) YJ: CK + 4 kg·ha^−1^ microbial organic fertilizer. This experimental design followed the method of Hou et al. [21]. This silage experiment adopted a 2 × 3 factorial design, including two fermentation methods—natural fermentation (CK) and inoculant addition (FJ)—and three fertilization treatments (CK, JJ, YJ). The detailed combinations are presented in Table 1. The natural fermentation group (CK) was sprayed with 200 mL of sterile water, whereas the treatment group (FJ) received 200 mL of sterile water containing 1% (*w*/*w*) silage inoculant. Each treatment was replicated five times and fermented for 30 days at room temperature (18–33 °C) in one-way valve bags. On day 0 and day 30, three bags per treatment were randomly sampled for analysis.

### 2.2. Determination of Indicators

For dry matter (DM) content determination, silage samples (150 g each) were dried at 65 °C for 48 h in triplicate. Starch content was measured using a commercially available ELISA kit (Enzymatic Method, Microplate/96-well) from China Shanghai Bioengineering Co., Ltd. (Shanghai, China). The contents of neutral detergent fiber (NDF) and acid detergent fiber (ADF) were determined according to the method described by González-García et al. [22]. Crude protein (CP) content was analyzed using the Kjeldahl method [23]. Ether extract (EE) content was assessed following the procedure of Hawu et al. [24]. The pH value was measured with a pH meter. Lactic acid (LA) content was determined as per the method of Tian et al. [25].

### 2.3. 16S rDNA Sequencing

Total genomic DNA was extracted from the samples using the E.Z.N.A.^®^ soil DNA Kit (Omega Bio-tek, Norcross, GA, USA). The DNA quality was assessed by 1% agarose gel electrophoresis and quantified using a NanoDrop 2000 spectrophotometer (Thermo Fisher Scientific, Wilmington, DE, USA). The V3–V4 hypervariable region of the bacterial 16S rRNA gene was then amplified from the qualified DNA templates with barcoded primers 338F and 806R. The PCR reactions (20 μL) contained 10 ng of template DNA, forward and reverse primers, and PCR master mix. The amplification protocol was as follows: initial denaturation at 95 °C for 3 min; 30 cycles of denaturation (95 °C for 30 s), annealing (55 °C for 30 s), and extension (72 °C for 30 s); followed by a final extension at 72 °C for 10 min. Following purification, the amplification products were quantified using a Qubit 4.0 fluorometer (Thermo Fisher Scientific, Wilmington, DE, USA). The purified products were pooled in equal amounts and sequenced on the Illumina MiSeq platform (Illumina, San Diego, CA, USA) with 250-bp paired-end reads. The raw sequencing reads were processed using fastp (v0.19.6) for quality control and FLASH (v1.2.11) for assembly. Subsequently, operational taxonomic units (OTUs) were clustered at a 97% similarity threshold, and chimeric sequences were removed using UPARSE (v11). On average, approximately 60,000 high-quality valid sequences were obtained per sample.

### 2.4. Statistical Analysis

Data are presented as mean ± standard deviation. All statistical analyses were performed using SPSS 22.0. A two-way repeated-measures ANOVA was applied to assess the main effects of the silage inoculant (between-subjects fixed effect), fertilization treatment (within-subjects fixed effect), and their interaction on the silage parameters, with subjects (e.g., silage replicates or experimental units) treated as a random factor. Prior to the main analysis, the normality of residuals was confirmed using Shapiro–Wilk tests (all *p* > 0.05), and the sphericity assumption was evaluated using Mauchly’s test. Post hoc tests were conducted using the LSD method. The results were considered statistically significant at *p* < 0.05.

## 3. Results

### 3.1. Pre-Ensiling Chemical Composition of Fresh Whole-Plant Corn

The chemical composition of fresh whole-plant corn under different fertilization treatments before ensiling is shown in Table 2. The contents of dry matter (DM), starch, NDF, and ADF in the YJ group were 31.00%, 29.09%, 44.38%, and 22.18%, respectively, which were higher than those in the CK and JJ groups. The contents of CP and EE in the CK group were 7.80% and 5.83%, respectively, both higher than those in the JJ and YJ groups.

### 3.2. Effect of Silage Inoculant on the Quality of Whole-Plant Corn Silage Under Different Fertilization Treatments (30 Days)

The chemical composition and fermentation quality of whole-plant corn silage after 30 days are presented in Table 3. A significant interaction between the silage inoculant and fertilization treatment was observed for the contents of DM, CP, EE, LA, NDF, ADF, and starch (*p* < 0.001). Under the same fertilization treatment, the FJ group exhibited higher CP, EE, LA, and starch but lower DM, NDF, and ADF compared to the natural fermentation (CK). Within the FJ group, the YJ fertilization treatment resulted in the highest DM, starch, and CP contents, while the JJ fertilization treatment yielded the lowest pH, LA, NDF, and ADF contents, along with the highest EE content. Furthermore, different fertilization treatments resulted in significant differences in pH (*p* < 0.001), with the JJ group showing the lowest pH. Compared to the natural fermentation group (CK), the addition of silage inoculant (FJ) led to a decrease in pH, although there was no significant difference between the CK and FJ groups (*p* > 0.05).

### 3.3. Analysis of Microbial Diversity in Whole-Plant Corn Silage Treated with Silage Inoculant Under Different Fertilization Regimes

**Figure 1:**
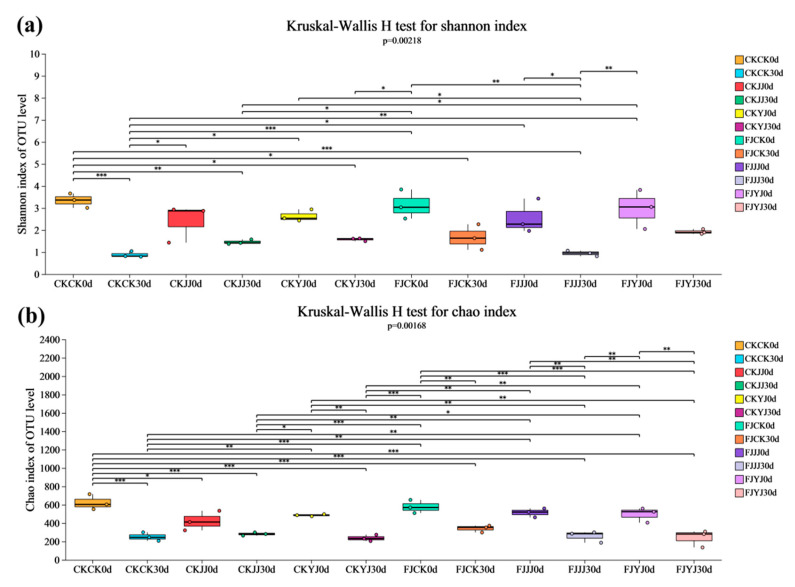
Analysis of bacterial alpha-diversity in whole-plant corn silage. The first CK in the set of experimental labels represents natural fermentation. FJ represents silage inoculants. The second CK in the set of experimental labels represents conventional fertilization, JJ represents liquid microbial inoculant + conventional fertilization and YJ represents microbial organic fertilizer + conventional fertilization treatment. * represents *p* < 0.05, ** represents *p* < 0.01, *** represents *p* < 0.001. The same below.

We analyzed alpha diversity across multiple groups using the Kruskal–Wallis H test (performing Welch’s (uncorrected) test followed by FDR correction). The Shannon and Chao1 indices were used to characterize the bacterial community’s alpha diversity in silage samples under different treatments on days 0 and 30 (Figure 1). The Shannon index revealed significant variations in bacterial diversity among the treatments (Figure 1a). The Shannon index differed significantly between day 0 and day 30 under both the CKCK (*p* < 0.001) and FJJJ (*p* < 0.05) treatments (Figure 1a). Furthermore, on day 30, a significant difference (*p* < 0.05) was observed between the CKCK and CKYJ treatments. The Chao1 index of the bacterial community is shown in Figure 1b. The Chao1 index decreased significantly from day 0 to day 30, with an extremely significant difference under the CKCK treatment (*p* < 0.001) and a highly significant difference across all silage inoculant treatments (*p* < 0.01) (Figure 1b).

**Figure 2:**
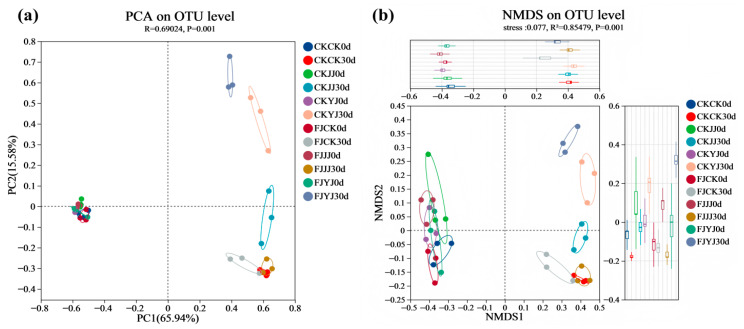
PCA and NMDS analysis of bacterial communities in whole-plant corn silage.

β-diversity was assessed using PCA and NMDS. For whole-plant corn silage, the first two principal components (PC1 and PC2) accounted for 65.94% and 15.5% of the total variance, respectively, in the PCA (Figure 2a). Clear separation was observed between day 0 and day 30 samples (R = 0.69024, *p* = 0.001). On day 30, the FJJJ and FJCK treatments formed distinct clusters from the FJYJ treatment, while the CKCK, CKJJ, and CKYJ treatments were also separated from one another (Figure 2a). A similar clustering pattern was confirmed by the NMDS analysis (Figure 2b).

**Figure 3:**
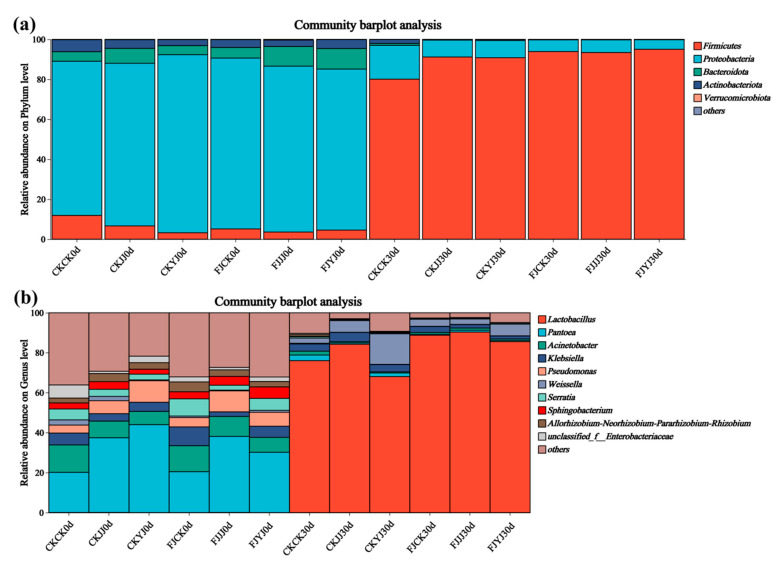
Bar plot of bacterial species composition at the phylum and genus levels in whole-plant corn silage. (**a**) is a phylum-level bar plot, and (**b**) is a genus-level bar plot.

The microbial composition at the phylum level shifted dramatically during ensiling (Figure 3a). Proteobacteria was the predominant phylum across all treatments on day 0, followed by Firmicutes. By day 30, Firmicutes became the dominant phylum, succeeding Proteobacteria, which exhibited a substantial decrease (Figure 3a). A similar taxonomic shift was observed at the genus level (Figure 3b). The initial community, dominated by *Pantoea*, *Acinetobacter*, *Klebsiella*, and *Pseudomonas*, was succeeded after 30 days of fermentation by a community overwhelmingly dominated by *Lactobacillus* and *Weissella* (Figure 3b).

**Figure 4:**
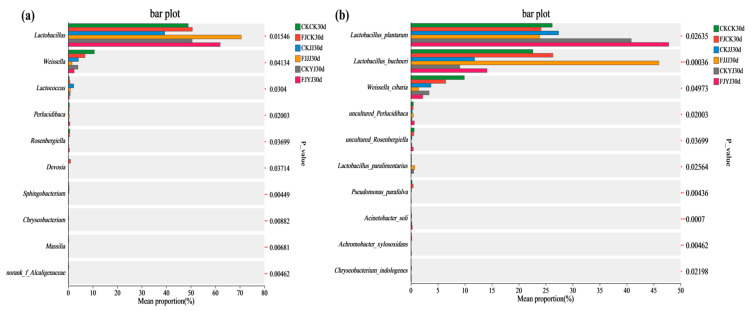
Analysis of differentially abundant bacterial taxa in whole-plant corn silage. (**a**) Genus-level differentially abundant taxa; (**b**) Species-level differentially abundant taxa. * represent *p* < 0.05, ** represent *p* < 0.01, *** represent *p* < 0.001

To investigate the specific effects of the silage inoculant on the microbial community in whole-plant silage under different fertilization regimes, a significance analysis of intergroup species differences was performed. We performed one-way ANOVA (using the Tukey–Kramer test followed by false discovery rate (FDR) correction) to analyze the significance of differences among treatment groups. Figure 4 reveals the average relative abundance of the microbial community and the significant differences among groups after 30 days of ensiling. At the genus level, *Lactobacillus* was the predominant genus. Although less abundant, genera such as *Sphingobacterium* and *Chryseobacterium* also exhibited significant intergroup differences (*p* < 0.01) (Figure 4a). At the species level, the dominant species were *Lactobacillus plantarum* and *Lactobacillus buchneri* (Figure 4b). The *Lactobacillus* group was the core dominant group, with obvious abundance differences among groups. Different treatment groups had a significant impact on species abundance and composition; meanwhile, there were also a small number of low-abundance but significantly different microbial groups.

**Figure 5:**
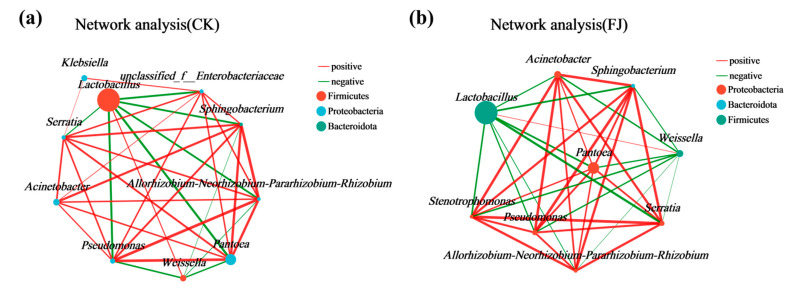
Genus-level co-occurrence network of bacterial taxa during the fermentation process of whole-plant corn silage. (**a**) shows the genus-level co-occurrence network of the control group, (**b**) shows the genus-level co-occurrence network of the silage inoculant group, where a larger circle indicates a higher abundance of the species.

Silage fermentation is an extremely complex microbial interaction process. To clarify the coexistence relationships and interactions of species during the fermentation process of whole-plant silage. This study constructed microbial co-occurrence networks based on a Spearman correlation analysis of the top 10 most abundant genera, illustrating species interactions during whole-plant silage fermentation under different fertilization treatments with silage inoculant (Figure 5). Under natural fermentation, the core dominant genus, *Lactobacillus*, was positively correlated with *Weissella* but negatively correlated with other genera, including *Pantoea* and *Pseudomonas* (Figure 5a). Specifically, *Weissella* exhibited significant negative correlations with both *Pantoea* and *Pseudomonas* (Figure 5a). Silage inoculant fermentation reduced the number of nodes in the co-occurrence network (Figure 5b). In this network, the dominant genus *Lactobacillus* exhibited a positive correlation with *Weissella* but negative correlations with other genera (Figure 5b). Notably, the number of dominant genera displaying negative correlations with both *Weissella* and *Lactobacillus* increased, including *Acinetobacter* and *Stenotrophomonas* (Figure 5b).

**Figure 6:**
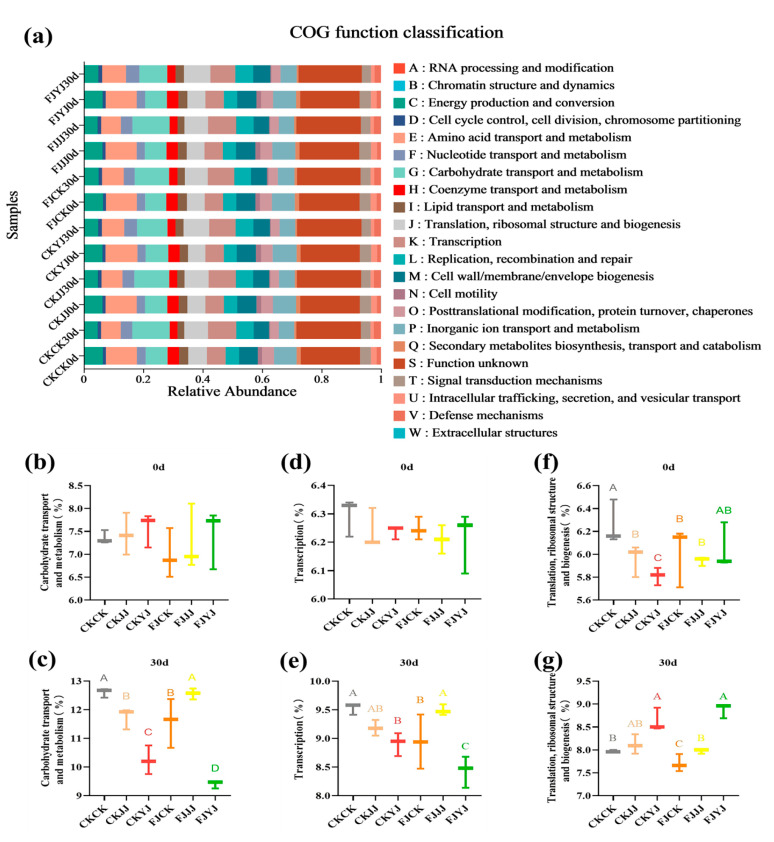
Statistical Analysis of Functional Classification of Species During the Whole-Plant Corn Silage Fermentation Process. Different capital letters indicate significant differences among treatments at the significance level of *p* < 0.05. (**a**) presents a statistical analysis of the functional classification of species during the silage fermentation process in the experimental and control groups (at 0 and 30 days of ensiling). (**b**) shows the functional analysis of carbohydrate transport and metabolism in the experimental and control groups at day 0 of ensiling, while (**c**) displays the corresponding analysis at day 30. (**d**) illustrates the translation function analysis for both groups at day 0 of ensiling, and (**e**) presents the same analysis at day 30. (**f**) provides the functional analysis of translation, ribosomal structure, and biogenesis for the experimental and control groups at day 0 of ensiling, and (**g**) shows the equivalent analysis at day 30.

Figure 6 presents the Clusters of Orthologous Groups (COG) functional classification of the microbial communities during the fermentation of whole-plant corn silage. Comparative analysis revealed minor differences in COG functional abundance between silage inoculant fermentation and natural fermentation across the different fertilization treatments. The most abundant functional categories included carbohydrate transport and metabolism, translation, ribosomal structure and biogenesis and transcription (Figure 6a). A marked increase in the abundance of specific COG functions was observed after 30 days of ensiling, particularly in carbohydrate transport and metabolism, translation, ribosomal structure and biogenesis and transcription (Figure 6a). At day 0 of ensiling, the functional abundances for carbohydrate transport and metabolism and for translation were comparable between the silage inoculant and natural fermentation treatments (Figure 6b,d). The CKCK treatment registered the highest abundances for biogenesis and transcription functions (*p* < 0.05) (Figure 6f). By day 30 of ensiling, a marked divergence in functional profiles was observed among the treatment groups. Regarding carbohydrate transport and metabolism, the FJJJ group exhibited the highest relative abundance, whereas the FJYJ group showed the lowest (Figure 6c). A similar pattern was observed for the translation function (Figure 6e). In contrast, for the combined functions of translation, ribosomal structure, and biogenesis, the FJYJ group registered the highest abundance, and the FJCK group the lowest (Figure 6g).

### 3.4. Correlation Between Dominant Bacterial Communities and Key Nutritional Indicators in Whole-Plant Corn Silage Treated with Silage Inoculant Under Different Fertilization Regimes

**Figure 7:**
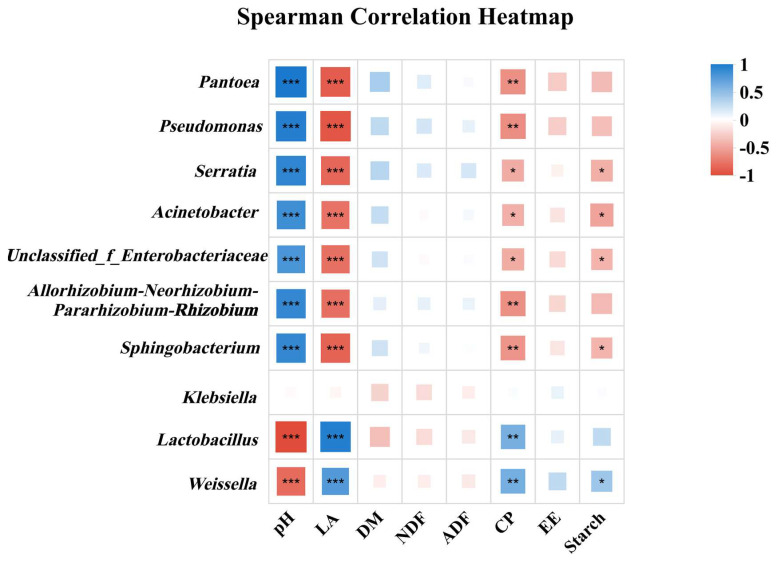
Correlation between dominant bacterial species and nutritional indicators in whole-plant corn silage. Blue color indicates positive correlations, while red color indicates negative correlations. * represents *p* < 0.05; ** represents *p* < 0.01; *** represents *p* < 0.001.

To investigate the relationship between the microbial community and fermentation quality in whole-plant silage, a Spearman correlation analysis was performed between the dominant bacterial species and key nutritional indicators (Figure 7). None of the dominant species showed a significant correlation with the contents of DM, NDF, ADF, or EE (*p* > 0.05). Similarly, *Klebsiella* was not significantly correlated with any of the measured nutritional parameters. *Lactobacillus* and *Weissella* demonstrated a negative correlation with pH (*p* < 0.001). In contrast to *Klebsiella*, the other nine bacterial genera showed a positive correlation with pH (*p* < 0.001). Regarding fermentation products, LA and CP contents were positively correlated with *Lactobacillus* and *Weissella* (*p* < 0.01). In contrast, these nutritional indicators were negatively correlated with most other genera (*p* < 0.05). Starch content was negatively associated with *Serratia*, *Acinetobacter*, *unclassified_f_Enterobacteriaceae*, and *Sphingobacterium* (*p* < 0.05), while it was positively associated with *Weissella* (*p* < 0.05).

## 4. Discussion

Dry matter (DM), crude protein (CP), neutral detergent fiber (NDF), acid detergent fiber (ADF), and lactic acid (LA) serve as key indicators for evaluating feed quality [26]. The contents of ADF, NDF, starch, and DM in the JJ and YJ groups were significantly higher than those in the CK group, while the contents of CP and EE were the highest in the CK group (Table 1). This may be attributed to the Bacillus subtilis present in both the liquid bacterial inoculant and the microbial organic fertilizer, which promoted plant growth and nutrient accumulation [27]. The silage inoculant group (FJ) had a significantly lower DM content than the natural fermentation group (CK), likely due to microbial consumption of nutrients during reproduction and metabolism [28]. A study by Carlos Eduardo et al. found that the addition of *Bacillus subtilis* in a hydroponic system can increase the leaf number and dry weight of lettuce, enhance the accumulation of nitrogen, calcium, magnesium, and sulfur in the leaves, and thereby improve lettuce yield and nutrient acquisition [29]. The silage inoculant group (FJ) had a significantly higher crude protein (CP) content than the natural fermentation group (CK). This difference was highly significant (*p* < 0.001). The likely reason is that the inoculant inhibited protein decomposition by aerobic microorganisms and pathogenic bacteria [30]. Consequently, the FJYJ group exhibited the highest CP content (Table 2). Lower contents of NDF and ADF improve the digestibility and palatability of fermented feed [31]. The contents of ADF and NDF were significantly lower in the silage inoculant group (FJ) than in the natural fermentation group (CK) (*p* < 0.001). Furthermore, the FJJJ group showed the lowest levels of both fibers. The improvement in silage quality is likely achieved by the cellulase in the inoculant, which destroys the plant cell wall, decomposes cellulose into soluble sugars, and thereby promotes the proliferation of lactic acid bacteria and lactic acid production [16]. The silage inoculant group (FJ) exhibited a significantly higher lactic acid content than the natural fermentation group (CK) (*p* < 0.001). Among all treatments, the FJJJ group recorded the highest content. This indicates that the silage inoculant increased the lactic acid content, which enhances silage quality—a finding consistent with Jin et al. [32]. This increase in lactic acid consequently lowered the pH value, a fundamental parameter for evaluating silage quality [33]. In this study, there was no significant difference in pH value between the silage inoculant group (FJ) and the natural fermentation group (CK) (*p* > 0.05), and all pH values were lower than 4.2. This indicates that the pH value of silage materials without silage inoculant addition can also reach the level of high-quality silage. This may be because under well-sealed and compacted conditions, whole-plant corn can use its endogenous lactic acid bacteria to quickly reduce the pH, thereby maintaining good anaerobic conditions.

During ensiling, the Shannon and Chao1 indices decreased across all treatments, indicating a reduction in bacterial richness and diversity. This decline is attributed to the acidic, anaerobic environment created by lactic acid produced by dominant lactic acid bacteria, which inhibits the growth of other microorganisms [34]. Beta diversity analysis (PCA and NMDS) confirmed that fermentation significantly altered the microbial community structure, with clear separation between the initial (day 0) and final (day 30) stages. At the end of silage (day 30), the analysis further revealed a clear separation among treatments within the natural fermentation group, indicating that pre-applied fertilization influenced the final microbial diversity. Most notably, the distinct clustering between the natural fermentation and silage inoculant groups directly demonstrates that the inoculant addition effectively reshaped the microbial community [35]. The silage process depends on the interaction of various bacteria, and the bacterial community structure directly affects silage quality [36]. To further elucidate the impact of silage inoculant application on the bacterial community composition in whole-plant silage, we assessed the composition and temporal dynamics of the bacterial community at both the phylum and genus levels. Throughout the ensiling process, the relative abundance of Lactobacillus progressively increased with the advancement of fermentation. The addition of silage inoculant can increase the abundance of the phylum Firmicutes and the genus *Lactobacillus* in silage corn feed under different fertilization treatments, and this result is consistent with the findings of Du et al. [37]. *Lactobacillus* is a type of lactic acid bacteria, which is the main bacterial group that inhibits aerobic bacteria. It produces lactic acid under anaerobic conditions, inhibits feed spoilage and the loss of nutrients, and improves the aerobic stability of silage [38]. Overall, the FJJJ treatment resulted in the highest relative abundance of *Lactobacillus* in whole-plant corn silage. This indicates that applying a silage inoculant following the JJ treatment can further optimize the silage process by enhancing lactic acid bacterial activity, leading to improved fermentation conditions and final feed quality. Significance analysis of species differences revealed that the silage inoculant significantly shaped distinct microbial communities under different fertilization treatments, altering both their abundance and composition [39]. Results of microbial network analysis showed that the addition of silage inoculant reduced the number of co-occurrence network nodes, which is consistent with the study by Xu et al. [40]. Positive correlations were observed between the genus *Lactobacillu*s and the genus *Weissella*. They showed negative correlations with most other genera. This further confirms that *Lactobacillus* and *Weissella* are dominant genera in high-quality silage and play a positive regulatory role during the fermentation process. PICRUSt2 analysis showed that the silage inoculant significantly enriched certain microbial functions under different fertilization treatments. Notably, carbohydrate metabolism was enhanced, which is closely related to nutrient transformation in silage [41]. Furthermore, this result also indicates that lactic acid bacteria proliferate extensively in the anaerobic environment, and their metabolic activities are significantly enhanced, thereby increasing the carbohydrate metabolic pathways [42]. This predicted functional enhancement is directly corroborated by the results presented in our Table 3: the FJ group exhibited a lower pH and a higher lactic acid content. This indicates that the enhanced functionality was directly translated into more efficient lactic acid fermentation, thereby more rapidly establishing a stable acidic environment that suppresses undesirable microorganisms. Furthermore, these results indicate that lactic acid bacteria proliferated substantially in the anaerobic environment and exhibited significantly elevated metabolic activity, which may be attributed to a synergistic enhancement of fundamental cellular functions such as transcription and translation.

Microorganisms exert influence on silage fermentation through their metabolic activities, thereby regulating the fermentation process and consequently affecting feed quality [43]. A positive correlation was observed between *Lactobacillus* and LA content, alongside a negative correlation with pH, a finding that aligns with the research by Peng et al. [44]. This is attributed to the competitive advantage of *Lactobacillus* during fermentation, which enables it to effectively outcompete other microorganisms for nutrients, thereby promoting efficient LA synthesis [45]. Furthermore, the positive correlation between *Lactobacillus* and CP content in the whole-plant silage further underscores its critical role in regulating silage quality. Furthermore, in this study, *Pantoea*, *Acinetobacter*, and *Serratia* were identified as dominant bacteria at the initial stage (day 0) of ensiling. However, they did not contribute positively to the fermentation process [46].

## 5. Conclusions

The results of this study demonstrate that the use of a silage inoculant (*Lactiplantibacillus plantarum* (60%), *Lactiplantibacillus buchneri* (30%), *Lactiplantibacillus casei* (5%), and cellulase (5%)) under the three fertilization treatments (CK, JJ, and FJ) enhanced the nutritional composition and fermentation parameters of whole-plant corn silage. Furthermore, the addition of the silage inoculant during the ensiling of whole-plant corn promoted the proliferation of lactic acid bacteria, suppressed undesirable microorganisms, and ultimately improved the quality of the silage. Therefore, the integration of an appropriate fertilization regime with a silage inoculant effectively enhances the quality of whole-plant corn silage. This study provides a new approach for the production of high-quality whole-plant corn silage. Future studies should explore more precision-controlled fertilization methods and the effects of different fermentation conditions on improving nutritional properties and microbial metabolites during the silage process, with the aim of achieving more efficient feed production.

## Figures and Tables

**Table 1:** 2 × 3 two-factor experimental design adopted for the silage experiment.

Ensilage Experimental Design Combinations	
CKCK	natural fermentation + conventional fertilization
CKJJ	natural fermentation + liquid microbial inoculant + conventional fertilization
CKYJ	natural fermentation + microbial organic fertilizer + conventional fertilization
FJCK	silage inoculants + conventional fertilization
FJJJ	silage inoculants + liquid microbial inoculant + conventional fertilization
FJYJ	silage inoculants + microbial organic fertilizer + conventional fertilization

CK denotes natural fermentation, whereas FJ indicates fermentation with a silage inoculant. The abbreviations CK (conventional fertilization), JJ (liquid microbial inoculant + conventional fertilization), and YJ (microbial organic fertilizer + conventional fertilization) refer to three distinct fertilization treatments.

**Table 2:** Chemical composition of whole-plant corn prior to ensiling.

Item	Treatment			*p*-Value
	CK	JJ	YJ	
DM%	28.00 ± 0.05	30.00 ± 0.03	31.00 ± 0.03	<0.001
Starch%	27.95 ± 0.08	28.94 ± 0.10	29.09 ± 0.03	<0.001
CP%	7.80 ± 0.01	7.14 ± 0.06	7.76 ± 0.01	<0.001
EE%	5.83 ± 0.01	5.81 ± 0.02	5.21 ± 0.02	<0.001
NDF%	33.24 ± 0.0	39.39 ± 0.86	44.38 ± 0.06	<0.001
ADF%	16.02 ± 0.15	18.17 ± 0.04	22.18 ± 0.06	<0.001

The abbreviations CK (conventional fertilization), JJ (liquid microbial inoculant + conventional fertilization), and YJ (microbial organic fertilizer + conventional fertilization) correspond to three distinct fertilization treatments. Statistical significance was defined at a level of *p* < 0.05. DM: dry matter; NDF: neutral detergent fiber; ADF: acid detergent fiber; CP: crude protein; EE: ether extract.

**Table 3:** Chemical composition and fermentation quality of whole-plant corn silage after 30 days.

Item	CK (Natural Fermentation)			FJ (Silage Inoculants)			*p*-Value		
	CK	JJ	YJ	CK	JJ	YJ	A	T	A × T
DM%	29.68 ± 1.02	26.43 ± 1.21	33.54 ± 0.14	28.54 ± 0.81	27.58 ± 0.71	31.55 ± 0.88	<0.001	<0.001	<0.001
pH%	3.72 ± 0.01	3.62 ± 0.04	3.80 ± 0.03	3.76 ± 0.02	3.61 ± 0.01	3.67 ± 0.01	0.091	<0.001	0.032
LA%	7.90 ± 0.02	7.63 ± 0.03	7.82 ± 0.02	7.94 ± 0.07	7.97 ± 0.03	7.94 ± 0.02	<0.001	0.004	0.009
Starch%	28.00 ± 0.16	29.54 ± 0.13	29.87 ± 0.13	30.53 ± 0.35	31.01 ± 0.05	33.26 ± 0.04	<0.001	<0.001	0.001
CP%	8.48 ± 0.04	7.63 ± 0.04	8.11 ± 0.02	8.42 ± 0.02	8.76 ± 0.01	9.03 ± 0.03	<0.001	<0.001	<0.001
EE%	6.60 ± 0.03	6.12 ± 0.04	6.69 ± 0.02	7.37 ± 0.06	8.87 ± 0.10	7.96 ± 0.02	<0.001	<0.001	<0.001
NDF%	37.31 ± 0.03	36.10 ± 0.49	40.06 ± 0.10	31.65 ± 0.08	31.62 ± 0.76	33.47 ± 0.09	<0.001	<0.001	<0.001
ADF%	21.36 ± 0.03	20.92 ± 0.16	20.25 ± 0.08	18.60 ± 0.10	15.21 ± 0.13	15.71 ± 0.04	<0.001	<0.001	<0.001

CK denotes natural fermentation, whereas FJ indicates fermentation with a silage inoculant. The abbreviations CK (conventional fertilization), JJ (liquid microbial inoculant + conventional fertilization), and YJ (microbial organic fertilizer + conventional fertilization) refer to three distinct fertilization treatments. Statistical significance was defined at *p* < 0.05. LA: lactic acid.

## Data Availability

The original contributions presented in this study are included in the article. Further inquiries can be directed to the corresponding authors.
